# Robust and Highly-Efficient Differentiation of Functional Monocytic Cells from Human Pluripotent Stem Cells under Serum- and Feeder Cell-Free Conditions

**DOI:** 10.1371/journal.pone.0059243

**Published:** 2013-04-03

**Authors:** Masakatsu D. Yanagimachi, Akira Niwa, Takayuki Tanaka, Fumiko Honda-Ozaki, Seiko Nishimoto, Yuuki Murata, Takahiro Yasumi, Jun Ito, Shota Tomida, Koichi Oshima, Isao Asaka, Hiroaki Goto, Toshio Heike, Tatsutoshi Nakahata, Megumu K. Saito

**Affiliations:** 1 Department of Clinilcal Application, Center for iPS Cell Research and Application, Kyoto University, Kyoto, Japan; 2 Department of Fundamental Cell Technology, Center for iPS Cell Research and Application, Kyoto University, Kyoto, Japan; 3 Department of Pediatrics, Kyoto University Graduate School of Medicine, Kyoto, Japan; 4 Department of Pediatrics, Yokohama City University Graduate School of Medicine, Yokohama, Japan; University of Tampere, Finland

## Abstract

Monocytic lineage cells (monocytes, macrophages and dendritic cells) play important roles in immune responses and are involved in various pathological conditions. The development of monocytic cells from human embryonic stem cells (ESCs) and induced pluripotent stem cells (iPSCs) is of particular interest because it provides an unlimited cell source for clinical application and basic research on disease pathology. Although the methods for monocytic cell differentiation from ESCs/iPSCs using embryonic body or feeder co-culture systems have already been established, these methods depend on the use of xenogeneic materials and, therefore, have a relatively poor-reproducibility. Here, we established a robust and highly-efficient method to differentiate functional monocytic cells from ESCs/iPSCs under serum- and feeder cell-free conditions. This method produced 1.3×10^6^±0.3×10^6^ floating monocytes from approximately 30 clusters of ESCs/iPSCs 5–6 times per course of differentiation. Such monocytes could be differentiated into functional macrophages and dendritic cells. This method should be useful for regenerative medicine, disease-specific iPSC studies and drug discovery.

## Introduction

Monocytic lineage cells, such as monocytes, macrophages and dendritic cells (DCs), are central to immune responses and play key roles in various pathological conditions. [1]–[2] Monocytes are the myeloid progeny of hematopoietic stem/progenitor cells [3]; they are a type of mononuclear cell circulating in the bloodstream and act as gatekeepers in innate immunity. While they replenish macrophages and DCs, monocytes themselves respond to various inflammatory stimuli by migrating into inflamed tissues, phagocytosing pathological small particles and producing proinflammatory cytokines and chemokines. Therefore, monocytes not only contribute to host defense against pathogenic microorganisms, but are closely associated with the pathogenesis of chronic sterile inflammation. [4] Macrophages reside in tissues and robustly phagocytose microorganisms and cellular debris. One of the important hallmarks of monocytic lineage cells is their functional plasticity. In response to cytokines and microbial products, macrophages polarize into functionally distinct M1 and M2 cells. [5] Classically activated M1 macrophages are induced by interferon-γ (IFNγ), while alternatively activated M2 macrophages can be induced by IL-4 and IL-13. [2], [5] M1 macrophages are generally characterized by high production of proinflammatory cytokines, while M2 are characterized by high production of anti-inflammatory cytokines. DCs are the most powerful antigen-presenting cells and have an indispensable role for the activation of T lymphocytes. Because of their ability to mediate communication between innate and acquired immunity, ex vivo expansion of DCs is expected to be a useful source of material for cancer immunotherapies, such as DC-based vaccines. [6]–[7] Moreover, recent reports of monocyte and/or DC deficiencies highlight the importance of understanding their development in humans. [8] However, there have been technical limitations for tracing the development of human monocytic cells, or for propagating them ex vivo.

Human embryonic stem cells (ESCs) and induced pluripotent stem cells (iPSCs) are undifferentiated pluripotent cells that can be propagated indefinitely. [9]–[11] The development of monocytic cells from these pluripotent cells is of particular interest because it would provide an unlimited source of these cells for clinical applications and the examination of disease pathologies. Although the methods for hematopoietic differentiation from ESCs/iPSCs using embryonic body or feeder co-culture systems have already been established, [12] these methods usually depend on xenogeneic feeder cells and/or animal- or human-derived serum, and therefore have a relatively poor-reproducibility. For instance, batch-to-batch variability of serum or feeder cells can influence the characteristics of *in vitro* differentiated DCs. [13] Here, we describe a novel serum- and feeder cell-free method that robustly and repetitively produces monocytic lineage cells from human ESCs/iPSCs.

## Materials and Methods

### Cell Culture

This study used human ESCs (cell line: KhES1) and iPSCs (cell line**s**: 201B7, 253G4, CIRA188Ai-W2, and CB-A11). [10], [14]–[15] 201B7, 253G4 [10] and CIRA188Ai-W2 [15] were previously described. A human ES cell line KhES1 was kindly provided by Dr. Norio Nakatsuji. Human iPS cell lines 201B7 and 253G4 were kindly provided by Dr. Shinya Yamanaka. CB-A11 was established from cord-blood mononuclear cells by using episomal vectors. [16] These ESCs/iPSCs were maintained on tissue culture dishes coated with growth factor-reduced Matrigel (Becton-Dickinson) in mTeSR1 serum-free medium (STEMCELL Technologies).

### Monocytic Lineage Cell Differentiation Method

**Figure 1:**
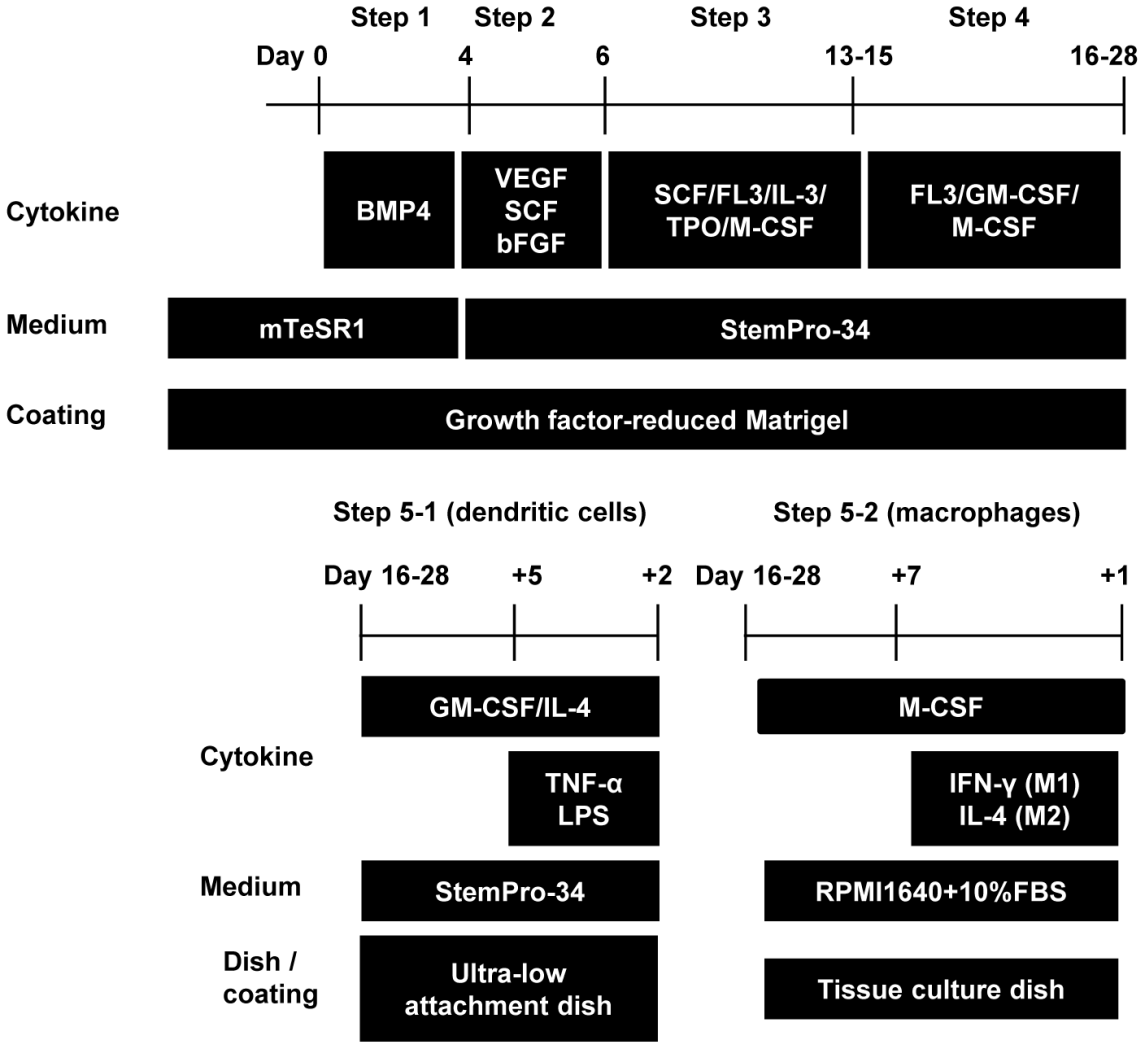
Protocol for monocytic lineage cell differentiation from human pluripotent stem cells. The protocol is composed of 5 steps. CD14-positive cells that are sorted between step-4 are differentiated into dendritic cells by step 5-1 or into macrophages by step 5-2. FL-3: Flt-3 ligand, TPO: Thrombopoietin.

The monocytic lineage differentiation protocol was modified from a previously established hematopoietic differentiation protocol (Figure 1). [17] The protocol consists of 5 sequential steps by which mature MPs and DCs are differentiated from human pluripotent cells in a stepwise manner. In the first step, primitive streak cells were induced from undifferentiated ESCs/iPSCs, which were then differentiated into hemangioblast-like hematopoietic progenitors in the second step. In step 3, expanded hematopoietic progenitors were committed towards initial myeloid differentiation, and then differentiated into the monocytic lineage in step 4. Finally, CD14^+^ monocytes were differentiated into either MPs or DCs in step 5. The cytokines used in this study were purchased from R&D systems.

#### Step 1: induction of primitive streak-like cells from undifferentiated human ES/iPS cells with BMP4

BMP4 is an important molecule for the initial stage of mesodermal commitment of pluripotent stem cells in vitro. [17] Undifferentiated ESCs/iPSCs colonies were disseminated onto a 100 mm culture dish coated with growth factor-reduced Matrigel in mTeSR1 medium at a density of about 30 colonies per dish. Individual colonies were grown to a diameter of approximately 1 mm (Day 0), and BMP4 (80 ng/mL) was added to the mTeSR1 medium.

#### Step 2: generation of KDR^+^CD34^+^ hemangioblast-like cells with VEGF, basic FGF and SCF

VEGF and SCF have been reported to be important cytokines for development of hemoangiogenic progenitors. [18]–[19] In this step, we also added basic FGF which enhances the development of mesodermal hematopoietic progenitors. [18], [20] The mTeSR1 medium was replaced by StemPro-34 serum-free medium (Gibco) containing 2 mM glutamax (Invitrogen) on day 4, and then was supplemented with the step-2 cytokine cocktail composed of VEGF (80 ng/mL), basic FGF (25 ng/ml), and SCF (100 ng/mL).

#### Step 3: generation of hematopoietic cells with hematopoietic cytokines

The cytokines in StemPro-34 medium were switched to the step-3 cytokine cocktail composed of SCF (50 ng/mL), IL-3 (50 ng/mL), TPO (Thrombopoietin) (5 ng/mL), M-CSF (50 ng/mL), and Flt-3 ligand (50 ng/mL), on day 6. Thereafter, the medium was changed on day 10.

#### Step 4: monocytic lineage-directed differentiation with Flt-3 ligand, GM-CSF and M-CSF

The cytokines in StemPro-34 medium were switched to the step-4 cytokine cocktail composed of Flt-3 ligand (50 ng/mL), GM-CSF (25 ng/mL), and M-CSF (50 ng/mL) on day 13–15. The medium was changed every 3–4 days. The CD14^+^ monocytic lineage-directed cell fraction in supernatant was positively sorted by autoMACS pro (Miltenyi Biotec) with CD14 MicroBeads (Miltenyi Biotec) on days 15–28.

#### Step 5: differentiation into DCs (step 5-1) and MPs (step 5-2) from CD14^+^ monocytic lineage-cells

CD14^+^ cells sorted by autoMACS pro (1.5×10^6^ cells per well in a 6-well plate with Ultra-Low Attachment Surface (CORNING)) were cultured in StemPro-34 medium supplemented with GM-CSF (25 ng/mL) and IL-4 (40 ng/mL), with a medium change 4 days later, for differentiation into DCs (step 5-1). LPS (100 ng/mL, InvivoGen) and TNFα (0.2 ng/mL) were added for the last 2 days of the 7 day DC differentiation culture to promote maturation of DCs. CD14^+^ cells (1.5×10^6^ cells per well in a 6-well tissue culture plate) were cultured in RPMI-1640 medium (Sigma) supplemented with 10% fetal bovine serum (FBS) and M-CSF (100 ng/mL) for 7 days with a medium change at day 4, for differentiation into macrophages (step 5-2). IFNγ (20 ng/ml) or IL-4 (20 ng/ml) was added for another day to promote differentiation into M1 or M2 macrophages, respectively.

### Flow Cytometric Analysis

Flow cytometric analysis data were collected using the MACS Quant™ Analyzer (Miltenyi Biotec) and then analyzed utilizing the FlowJo software package (Treestar). The following antibodies were purchased from BD Biosciences: CD11b-FITC, CD11c-APC, CD34-PE, CD40-PE, CD43-FITC, CD80-PE, CD83-PE, CD86-FITC, CD205-Alexa fluor 647, CD206-FITC, CD209-PE, HLA-ABC-FITC and HLA-DR-FITC. CD14-APC and CD45-APC antibodies were purchased from Beckman Coulter. CD163-APC antibody was purchased from R&D systems. KDR (CD309)-Alexa fluor 647 and CX3CR1-PE antibodies were purchased from Biolegend.

### May-Giemsa Staining and Esterase Staining

Cells were seeded onto glass slides by CYTOSPIN 4 (Thermo Scientific) and stained with May-Grunwald and Giemsa staining solution (MERCK) and Esterase staining solution (Muto pure chemicals) following the manufacturer’s instructions.

### RNA Extraction and RT-PCR Analysis

RNA samples were prepared using the RNeasy mini kit (Qiagen) following the manufacturer’s instructions. Typically, 500 ng of total RNA were subjected to reverse transcription (RT) with a Sensiscript-RT kit (Qiagen). RT-PCR was performed for the evaluation of the expression of monocytic lineage marker genes such as *PU.1, MAF, TLR4, CCL17* and *CCL18* using the primers in **Table S1**. [21]–[22] Peripheral blood monocyte-derived mature DCs/macrophages were generated from peripheral CD14^+^ monocytes using the step 5-1/5-2 cytokine cocktails in 10% FBS-supplemented RPMI-1640 for use as positive controls.

### Cytokine Assay

Concentrations of cytokines (IL-1β, IL-6, IL-10, IL-12p70 and TNFα) in supernatants were analyzed with FlowCytomix kits (Bender MedSystems) following the manufacturer’s instructions. The IL-1β, IL-6 and TNFα levels in the culture supernatants of pluripotent cell-derived monocytes (PS-Mo) were analyzed in three settings, (1) culture in RPMI-1640 medium supplemented with 10% FBS and LPS (100 ng/ml) for 4.5 hours, (2) as in (1) but with the addition of ATP (1 mM) for the last 30 min, (3) without LPS or ATP for 4.5 hours, to evaluate the production pattern of IL-1β in response to LPS plus ATP. [23].

The levels of IL-6, IL-10, IL-12p70 and TNFα in the supernatants of M1 or M2 macrophage culture were measured 24 hours after LPS (100 ng/ml) stimulation.

### Chemotaxis Assay

PS-Mo chemotaxis was evaluated using a trans-well migration assay with 8-µm pore size inserts (BD Biosciences). After CX3CL1 (fractalkine; R&D systems) was added to either the bottom or top of the chamber, serum-starved PS-Mo were loaded onto the inserts which were placed into 24-well plates containing RPMI-1640 and then incubated at 37°C for 5 hours. [24] Cell migration was measured by flow cytometry as previously reported: equivalent amounts of counting beads were added to each sample and the ratios of PS-Mo to the counting beads were calculated. [25].

### Antigen Uptake Assay

The antigen uptake capacity of monocytic lineage cells was evaluated as previously described. [26] Briefly, the cells were collected and stored on ice for 10 min. PS-Mo, pluripotent cell-derived immature DCs (PS-imDCs) and pluripotent cell-derived mature DCs (PS-mDCs) (5×10^4^ cells) were incubated with Ovalbumin Alexa fluor 488 Conjugate (Molecular Probes) at 0.1 mg/ml at 37°C or on ice for 45 min. Ice-cold FACS buffer was added in order to stop the reaction, samples washed twice, and the fluorescence intensity analyzed by flow cytometry.

### Mixed Leukocyte Reactions

Allogeneic naïve T cells (1×10^5^ cells per well) were purified from umbilical cord blood mononuclear cells using naïve CD4^+^ T cell isolation kits (Miltenyi Biotec) and then co-cultured with 40 Gy-irradiated stimulator cells (PS-Mo, PS-imDC, and PS-mDC) in 96-well round bottomed culture plates for 3–5 days. ^3^H-methylthymidine (25 uCi/ml, Moravek Biochemicals and Radiochemicals) was added to the culture medium of 10% FBS-supplemented RPMI-1640 for the last 16 hours. The cells were harvested onto a filter mat (Perkin Elmer) and the ^3^H methylthymidine uptake determined using a scintillation counter (MicroBeta TriLux, Perkin Elmer).

### Ethical Considerations

This study was approved by the Ethics Committee of Kyoto University and written informed consent was obtained from each healthy volunteer.

### Statistics

Data are presented as the mean ± S.D. and the statistical significance of the differences between cultures were evaluated by Student’s *t*-test.

## Results

### Differentiation of ESCs/iPSCs into Dendritic Cells and Macrophages via Monocyte-like Cells

**Figure 2:**
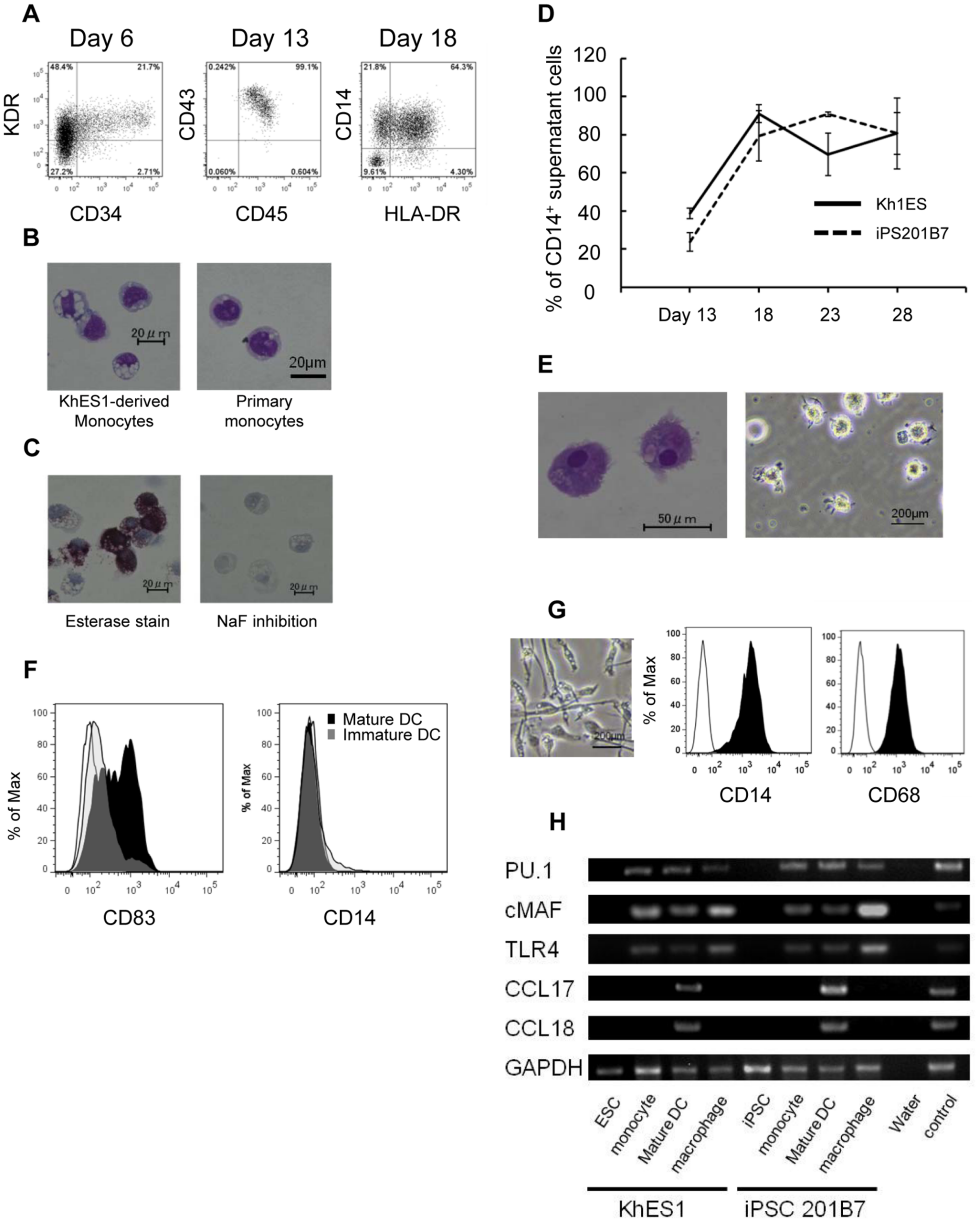
Phenotype analysis and gene expression pattern of monocytic lineage cells derived from pluripotent stem cells. (A) Flow cytometric analysis of monocytic lineage cells derived sequentially from pluripotent stem cells. An analysis of adherent cells on day 6 and supernatant cells on day 13 and 18 is shown. (B) May-Giemsa staining of CD14^+^ monocyte-like cells derived from KhES1 on day 16 (left) and primary human monocytes (right). (C) Esterase staining for CD14^+^ monocyte-like cells derived from KhES1 on day 16. (D) The percentage of CD14^+^ cells within the total floating cells derived from KhES1/iPS-201B7 was evaluated from day 13 to day 28. (E) May-Giemsa staining (left) and phase contrast image (right) of mature DCs derived from pluripotent stem cells. (F) Flow cytometric analysis of immature/mature DCs derived from pluripotent stem cells. (G) Phase contrast image and flow cytometric analysis of macrophages derived from pluripotent stem cells.(H) RT-PCR analysis of monocytic lineage cells derived from KhES1/iPS-201B7 clones for expression of monocytic lineage marker genes (*PU.1, c-MAF, TLR4, CCL17* and *CCL18*). Peripheral blood monocytes and peripheral blood monocyte-derived mature DCs were used as positive controls.(A–C, E–G) The data from KhES1-derived cells are shown as representative.

A KDR^+^CD34^+^ hemangioblast-like population was detected in adherent cell clusters on day 6 (steps 1,2), and around 95% of supernatant cells were CD43^+^CD45^+^ hematopoietic cells on days 13–15 (step 3; **Figure 2A**). [17] Floating cells were recovered every 3–4 days in step 4 **(Figure S1)**; the majority of these cells were CD14^+^ monocyte-like cells (**Figure 2A**). These pluripotent cell-derived monocytes (PS-Mo) were similar to peripheral blood monocytes in morphology (**Figure 2B**). PS-Mo are positive for Esterase staining which was inhibited by NaF (**Figure 2C**). The percentages of PS-Mo in floating cells were constantly about 50–90% between day 18–28 (**Figure 2D** **and Figure S2A**). The yield of PS-Mo per 100 mm culture dish starting with about 30 colonies was 1.3×10^6^±0.3×10^6^ at each step-4 medium exchange.

To derive DCs, PS-Mo were purified by magnetic sorting, and differentiated into CD14^−^CD83^−^ immature DCs (PS-imDCs) with the step 5-1 cytokine cocktail in 5 days (**Figure 2E**). PS-imDCs were stimulated with LPS and TNFα for an additional 2 days, which further differentiated them into CD14^−^CD83^+^ mature DCs (PS-mDCs) (**Figure 2F**). The differentiation efficiency of mature DCs from PS-Mo was comparable to that from primary monocytes (7.7%±0.9% vs. 16.5%±1.0%, p = 0.20, unpaired Student’s *t*-test). PS-Mo also had the potential to differentiate into macrophages (PS-MPs) with the step 5-2 cytokine cocktail. PS-MPs are morphologically comparable to primary monocyte-derived macrophages and they express typical surface markers such as CD14 and CD68 (**Figure 2G** **and Figure S3A,B**).

We confirmed that PS-Mo, pluripotent cell-derived DCs (PS-DCs), and PS-MPs expressed monocytic lineage-specific genes (**Figure 2H** **and Figure S2B**). [22], [27] Collectively, by using this protocol, sufficient numbers of monocytic cell lineage cells can be obtained from a small number of human ESCs/iPSCs.

### Functional Assays for Monocytes Derived from ESCs/iPSCs

**Figure 3:**
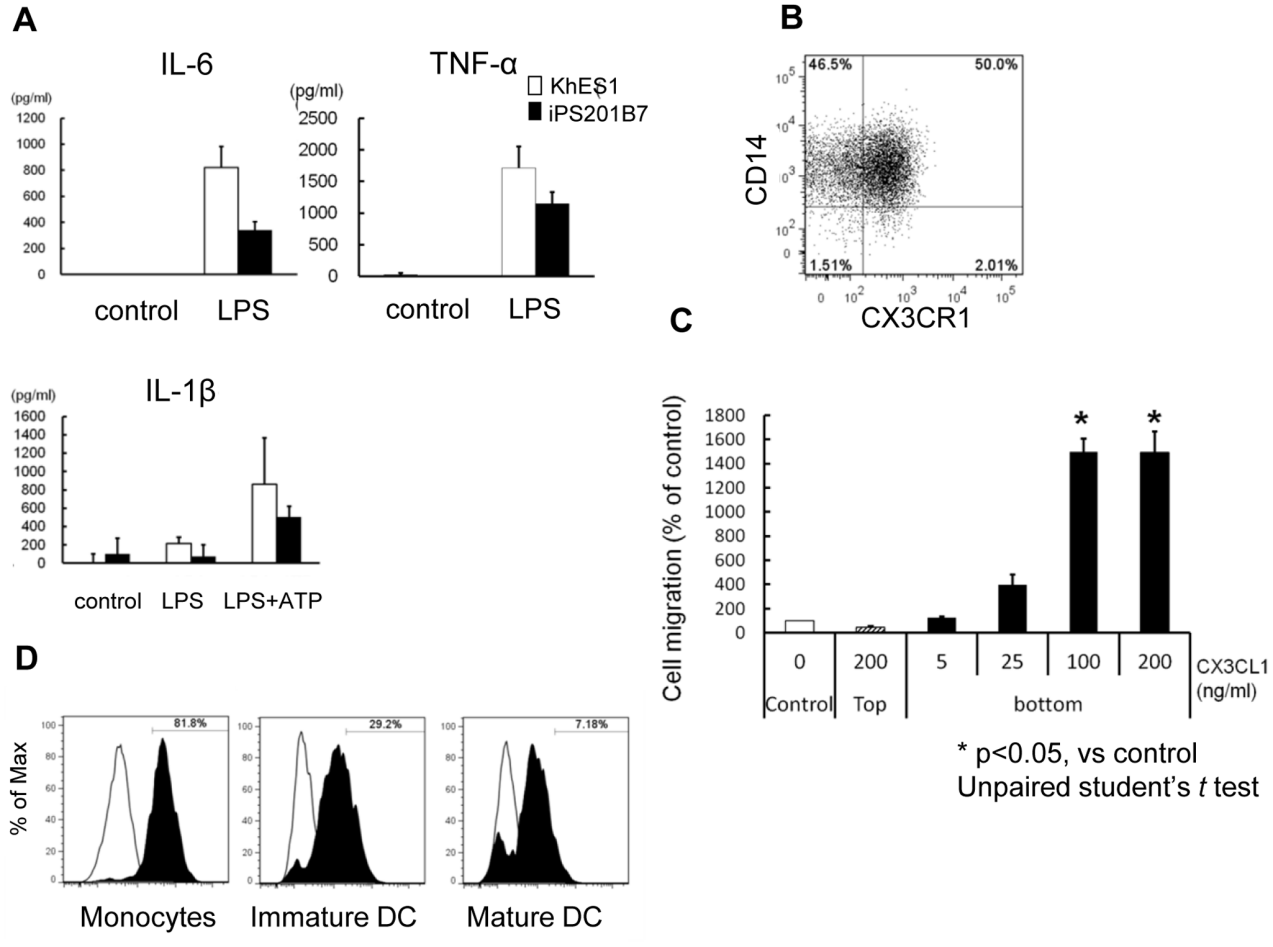
Functional assays for monocytes derived from pluripotent stem cells. (A) The levels of IL-6 and TNFα in supernatants of PS-Mo culture medium 4 hours after LPS stimulation. The levels of IL-1β were measured 4 hours after LPS stimulation with/without an additional 30-minute ATP stimulation. (B) Flow cytometric analysis of CX3CR1 on PS-Mo. (C) Chemotaxis assay of PS-Mo for CX3CL1 (fractalkine) using a trans-well migration assay. After the addition of CX3CL1 into either the bottom or top of the trans-well chamber, PS-Mo were applied and incubated for 5 hours at 37°C. (D) Antigen uptake was evaluated in monocytes, immature DCs and mature DCs derived from pluripotent stem cells by examining the fluorescence intensity of Alexa fluor 488-conjugated ovalbumin 45 minute after incubation at 37°C (black). Control samples (white) were kept on ice. (B–D) The data of KhES1-derived cells are shown as representative. PS-Mo: monocyte derived from pluripotent stem cells.

Next, we evaluated the functional activity of pluripotent cell-derived monocytic lineage cells. PS-Mo robustly produced the pro-inflammatory cytokines IL-6 and TNFα after LPS stimulation (**Figure 3A** **, Figure S3C**). Secretion pattern of IL-1β from PS-Mo with two stepwise signals LPS and ATP were similar to primary monocytes (**Figure 3A** **, Figure S3D**). [23], [28].

PS-Mo expressed CX3CR1, implying chemotactic responses to CX3CL1 (fractalkine) (**Figure 3B**). PS-Mo migration in trans-well assays increased with increasing doses of CX3CL1 in the lower compartment of the chamber (**Figure 3C**). This phenomenon was not due to chemokinesis, but chemotaxis, because CX3CL1 in the top compartment could not induce PS-Mo migration into the lower compartment of the chamber. [24] We next compared the antigen uptake ability of PS-Mo, PS-imDCs, and PS-mDCs by incubating them with Ovalbumin Alexa fluor 488 Conjugate. [26] PS-Mo had the highest ability to take up antigen and as DCs matured they lost their ability to endocytose antigens (**Figure 3D**).

### Functional Assays for DCs Derived from ESCs/iPSCs

**Figure 4:**
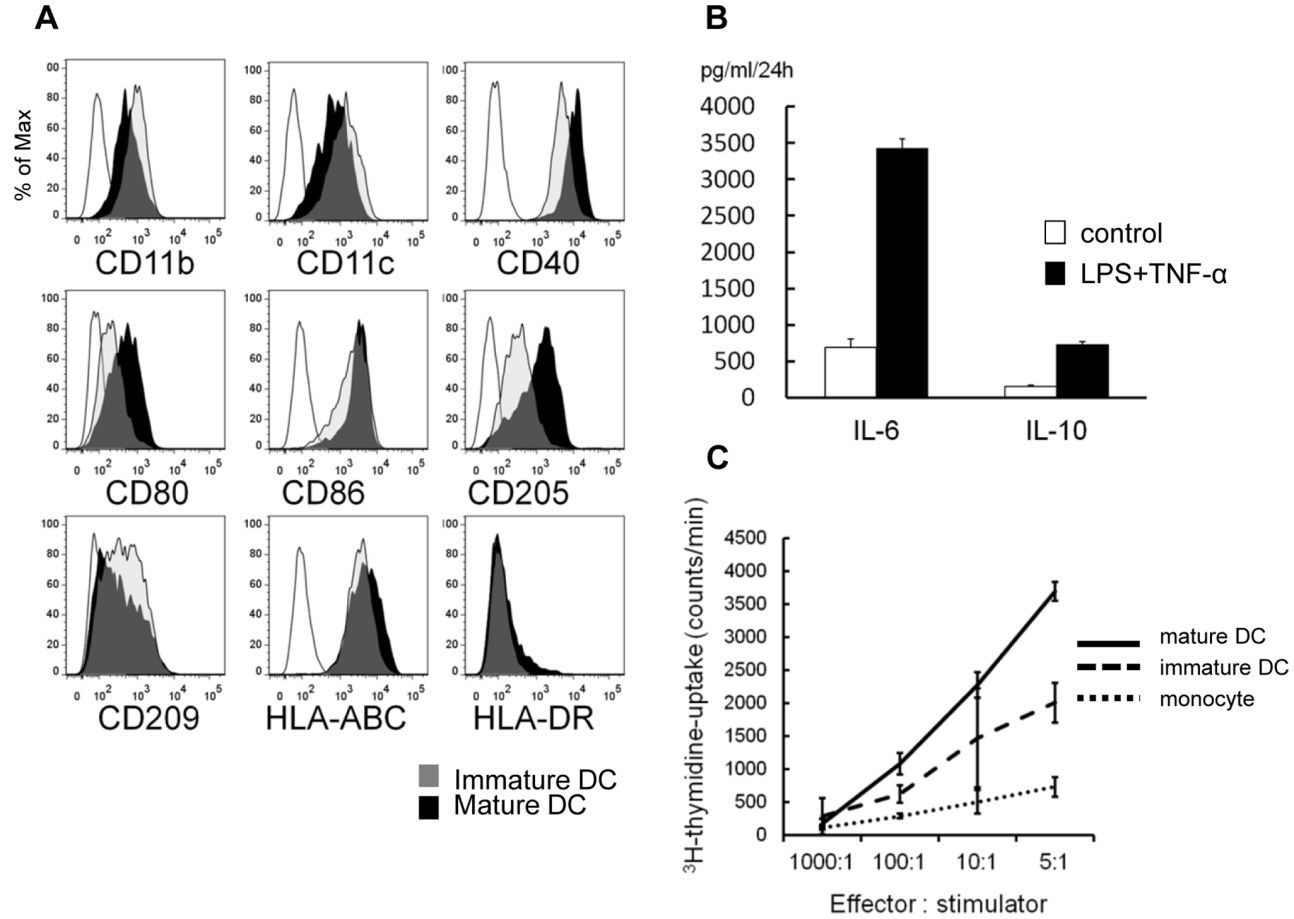
Functional assays for dendritic cells derived from pluripotent stem cells. (A) Flow cytometric analysis of immature/mature DCs derived from pluripotent stem cells. (B) The levels of IL-10 and TNFα in supernatants of culture medium with PS-DCs 24 hours after LPS stimulation. (C) The proliferation of allogeneic naïve T cells (1×10^5^ cells per well) co-cultured with 40 Gy-irradiated stimulator cells for 3 days was evaluated. The proliferation of naïve T cells in the last 16 hours was measured by ^3^H-thymidine uptake. (A–C) The data of KhES1-derived cells are shown as representative.

For evaluating functions of PS-DCs, we first confirmed that patterns of expression of cell surface markers on PS-imDCs/mDCs were comparable to those on primary dendritic cells (**Figure 4A** **, Figure S4A**). When stimulated with LPS and TNFα, PS-DCs also produced almost comparable amounts of pro-inflammatory and anti-inflammatory cytokines (**Figure 4B** **, Figure S4B**).

To test the ability of PS-DCs to activate naïve T cells, we next co-cultured allogeneic naïve T cells with PS-DCs and PS-Mo. As shown in Figure 4C, PS-mDCs had the most potent capacity to stimulate allogeneic T cell proliferation and this dose-response relationship was comparable to that observed with PB-DCs (Figure S4C).

### Functional Assays for Macrophages Derived from ESCs/iPSCs

**Figure 5:**
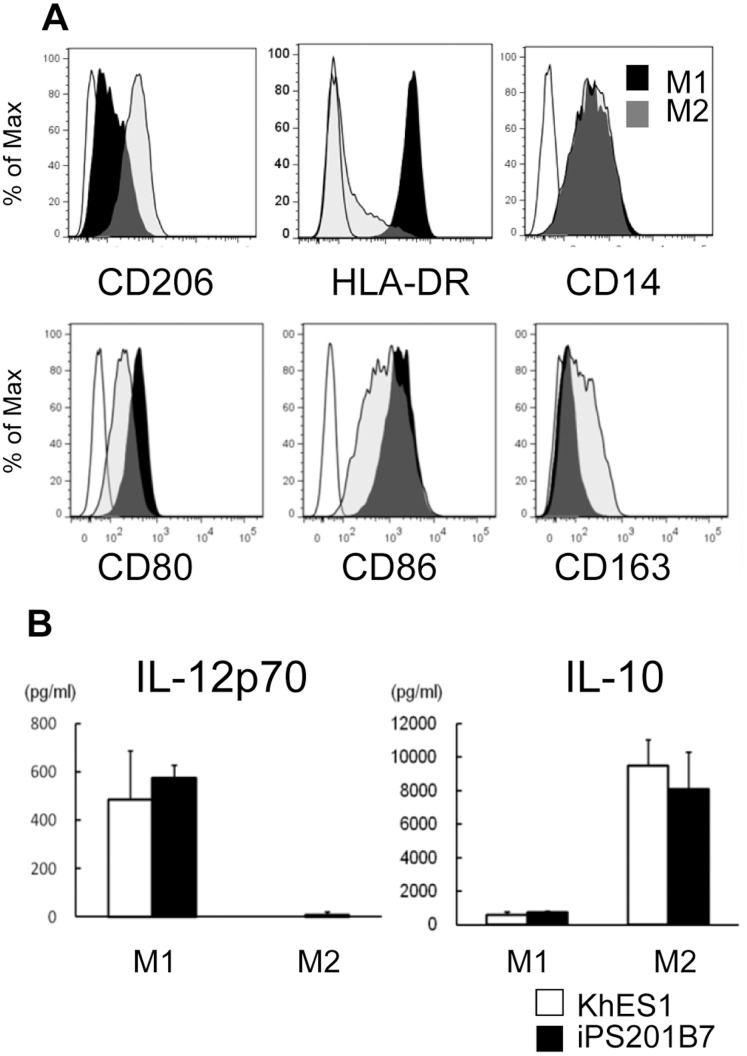
Functional assays for M1/M2 macrophages derived from pluripotent stem cells. (A) Flow cytometric analysis of M1/M2 macrophages derived from pluripotent stem cells. (B) The levels of IL-12p70 and IL-10 in supernatants of culture medium with M1/M2 macrophages derived from pluripotent stem cells 24 hours after LPS stimulation. The data of KhES1-derived cells are shown as representative.

Using this technique, we obtained morphologically typical macrophage-like cells that adhered firmly to the culture dish. To test whether these PS-MPs possessed functional plasticity like primary macrophages, we tried to polarize them into M1 or M2 state by treating them with IFNγ or IL-4, respectively. PS-MPs exhibited typical surface markers that were characteristic of primary M1 or M2 macrophages (**Figure 5A** **, Figure S5A**). The M1 cytokine pattern is typically IL-12^high^ and IL-10^low^, whereas the M2 pattern is IL-12^low^ and IL-10^high^. [5] Pluripotent cell-derived M1 and M2 macrophages (PS-M1/M2) also exhibited cytokine profiles that were comparable to those generated from primary monocytes (**Figure 5B** **, Figure S5B**).

## Discussion

We have established a novel differentiation system for monocytic cells from human ES and iPS cells. Since macrophages and dendritic cells are usually obtained *in vitro* from monocytes, the most important point of the evaluation is to establish whether monocytes differentiated from ESCs/iPSCs are functionally comparable to primary monocytes. In several functional assays, PS-Mo indeed proved to be comparable to primary monocytes, and importantly, PS-DCs and PC-MPs from PS-Mo were also functionally comparable to their primary counterparts.

Although complete M1/M2 macrophage polarization still requires aserum-containing medium, the present results prove that the current method can precisely manipulate macrophages that have the potential to differentiate into M1/M2 macrophages. The cytokine profiles of PS-M1/M2 were also comparable to those of primary M1/M2 macrophages. The expression patterns of surface markers in PS-DCs after LPS stimulation and of PS-MPs after M1/M2 polarization were almost identical to those of DCs/MP derived from primary monocytes. However, the level of IL-10 in PS-DCs after stimulation was higher than that in primary DCs and the expression levels of HLA-DR in PS-DCs/MP were low in comparison with those in DCs/MP derived from primary monocytes. Therefore, further improvement of culture conditions such as the use of a modified medium and cytokine cocktail will be needed.

Several embryonic body methods and feeder cell co-culture methods for PS-DCs/MP differentiation have already been reported. [7], [27], [29]–[30] These methods show relatively poor-reproducibility because of the use of xenogeneic feeder cells and/or serum. In an earlier report which describes a protocol that can derive macrophages and dendritic cells from human iPSCs in feeder- and serum-free manner, [7] the authors did not fully characterize the monocytes and noted that PS-DCs/MP were generated only from two of the five iPSC clones tested. The current culture system simply propagated progenitor cells in 2-dimensional cultures without passage or sorting, and floating PS-Mo and PS-DCs/MP could be obtained repetitively from all five ESC/iPSC clones tested (**Figure S2 and S6**). These monocytic cells derived from disease- or patient- specific iPSC would be useful tools for the examination of disease pathologies and for drug discovery in immunological disorders such as autoimmune diseases, immunodeficiencies and autoinflammatory syndromes. However, even in our protocol, there are subtle clonal variations of timing of differentiation such as the day of step 3 to 4 switching which is determined by the emergence of CD43^+^CD45^+^ cells (day 13–15, data not shown). Fine adjustment of the protocol for each ESC/iPSC clone seemed to further improve the yield of monocytes.

iPSC technology is overcoming immunological and ethical concerns in regenerative medicine using human pluripotent cells. Furthermore, a number of disease-associated iPSCs generated from patients with immunological disorders have been reported. [15], [31]–[34] Because patient- or disease-specific iPS cells will be an important resource for unraveling human immunological disorders, a robust and simple hematopoietic differentiation system that can reliably mimic in vivo hematopoiesis is necessary for this purpose. Our simple and robust protocol to produce monocytic cells is therefore expected to be useful for regenerative medicine and studies of immunological disorders.

## Supporting Information

Figure S1 **Image of floating hematopoietic cells derived from iPS cells Phase contrast image of floating hematopoietic cells derived from iPS-201B7 at day 21 (step 4).**
(PDF)

Figure S2 **Phenotype analysis and gene expression pattern of monocytic lineage cells derived from 3 additional pluripotent stem cell lines.** (A) The percentage of CD14+ cells within the total floating cells derived from 3 iPSC clones (253G4, CIRA188Ai-W2, and CB-A11) was evaluated from day 13 to day 28. (B) RT-PCR analysis of monocytic lineage cells derived from 253G4, CIRA188Ai-W2, and CB-A11 clones for expression of monocytic lineage marker genes (c-MAF, TLR4, and CCL17). Peripheral blood monocytes and peripheral blood monocyte-derived mature DCs were used as positive controls.
(PDF)

Figure S3 **Characteristics of primary monocytes and macrophages.** (A) Phase contrast image and (B) flow cytometric analysis of macrophages derived from primary monocytes. (C) The levels of IL-6 and TNF-α in supernatants of primary monocyte culture medium 4 hours after LPS stimulation. (D) The levels of IL-1β were measured 4 hours after LPS stimulation with/without an additional 30-minute ATP stimulation.
(PDF)

Figure S4 **Characteristics and functional assays of dendritic cells derived from primary monocytes.** (A) Flow cytometric analysis of immature/mature DCs derived from primary monocytes. (B) The levels of IL-10 and TNF-α in supernatants of culture medium with primary-DCs 24 hours after LPS stimulation. (C) The proliferation of allogeneic naïve T cells (1×105 cells per well) co-cultured with 40 Gy-irradiated stimulator cells for 3 days was evaluated. The proliferation of naïve T cells in the last 16 hours was measured by 3H-thymidine uptake.
(PDF)

Figure S5 **Characteristics and functional assays of M1/M2 macrophages derived from primary monocytes.** (A) Flow cytometric analysis of M1/M2 macrophages derived from primary monocytes. (B) The levels of IL-12p70 and IL-10 in supernatants of culture medium with M1/M2 macrophages derived from primary monocytes 24 hours after LPS stimulation.
(PDF)

Figure S6 **Replication assays for 3 additional pluripotent stem cell lines.** (A) Phase contrast image (left) and May-Giemsa staining (right) of mature DCs derived from iPSC clones. (B) Phase contrast image of macrophages derived from iPSC clones. (C) Flow cytometric analysis of immature/mature DCs and macrophages derived from iPSC clones.
(PDF)

Table S1 **Primers for RT-PCR.**
(PDF)
