# Supplementary figures and images for: Robust and Highly-Efficient Differentiation of Functional Monocytic Cells from Human Pluripotent Stem Cells under Serum- and Feeder Cell-Free Conditions
Source: PLoS One. 2013 Apr 3;8(4):e59243. doi: 10.1371/journal.pone.0059243 (PMC3616072; doi:10.1371/journal.pone.0059243)

**Figure S1**

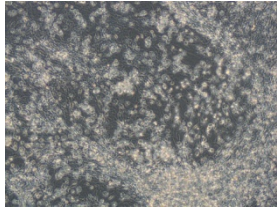

Supplement: Figure S1 — Image of floating hematopoietic cells derived from iPS cells Phase contrast image of floating hematopoietic cells derived from iPS-201B7 at day 21 (step 4). (PDF) [file pone.0059243.s001.pdf]

Figure S2

A

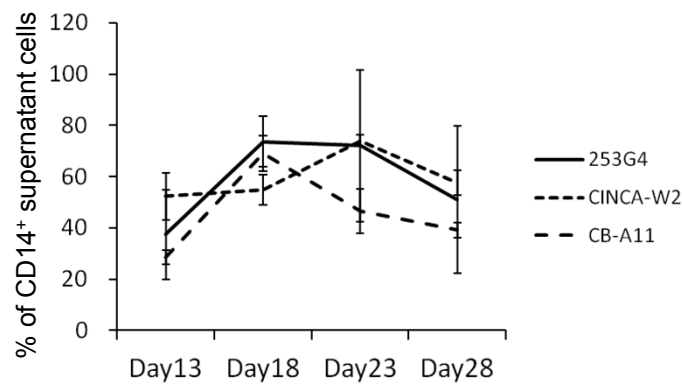

B

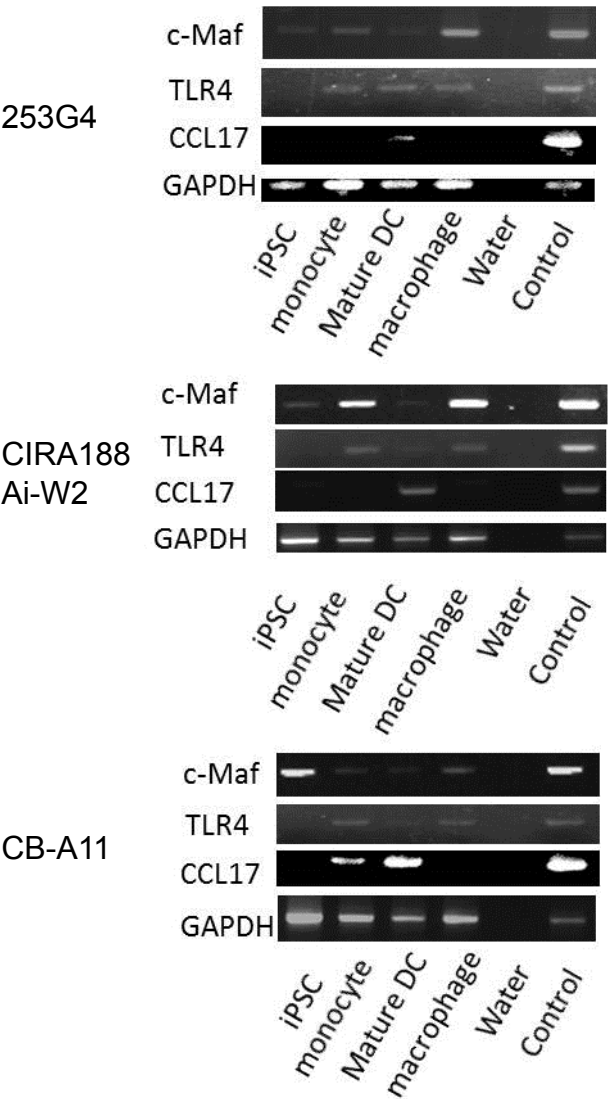

Supplement: Figure S2 — Phenotype analysis and gene expression pattern of monocytic lineage cells derived from 3 additional pluripotent stem cell lines. (A) The percentage of CD14+ cells within the total floating cells derived from 3 iPSC clones (253G4, CIRA188Ai-W2, and CB-A11) was evaluated from day 13 to day 28. (B) RT-PCR analysis of monocytic lineage cells derived from 253G4, CIRA188Ai-W2, and CB-A11 clones for expression of monocytic lineage marker genes (c-MAF, TLR4, and CCL17). Peripheral blood monocytes and peripheral blood monocyte-derived mature DCs were used as positive controls. (PDF) [file pone.0059243.s002.pdf]

Figure S3

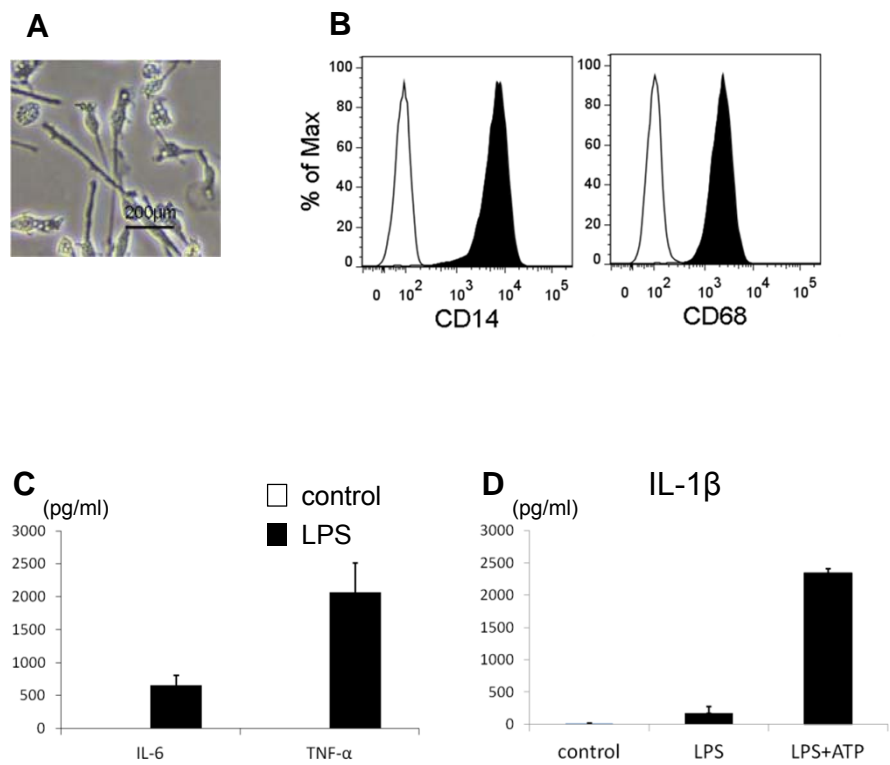

Supplement: Figure S3 — Characteristics of primary monocytes and macrophages. (A) Phase contrast image and (B) flow cytometric analysis of macrophages derived from primary monocytes. (C) The levels of IL-6 and TNF-α in supernatants of primary monocyte culture medium 4 hours after LPS stimulation. (D) The levels of IL-1β were measured 4 hours after LPS stimulation with/without an additional 30-minute ATP stimulation. (PDF) [file pone.0059243.s003.pdf]

Figure S4

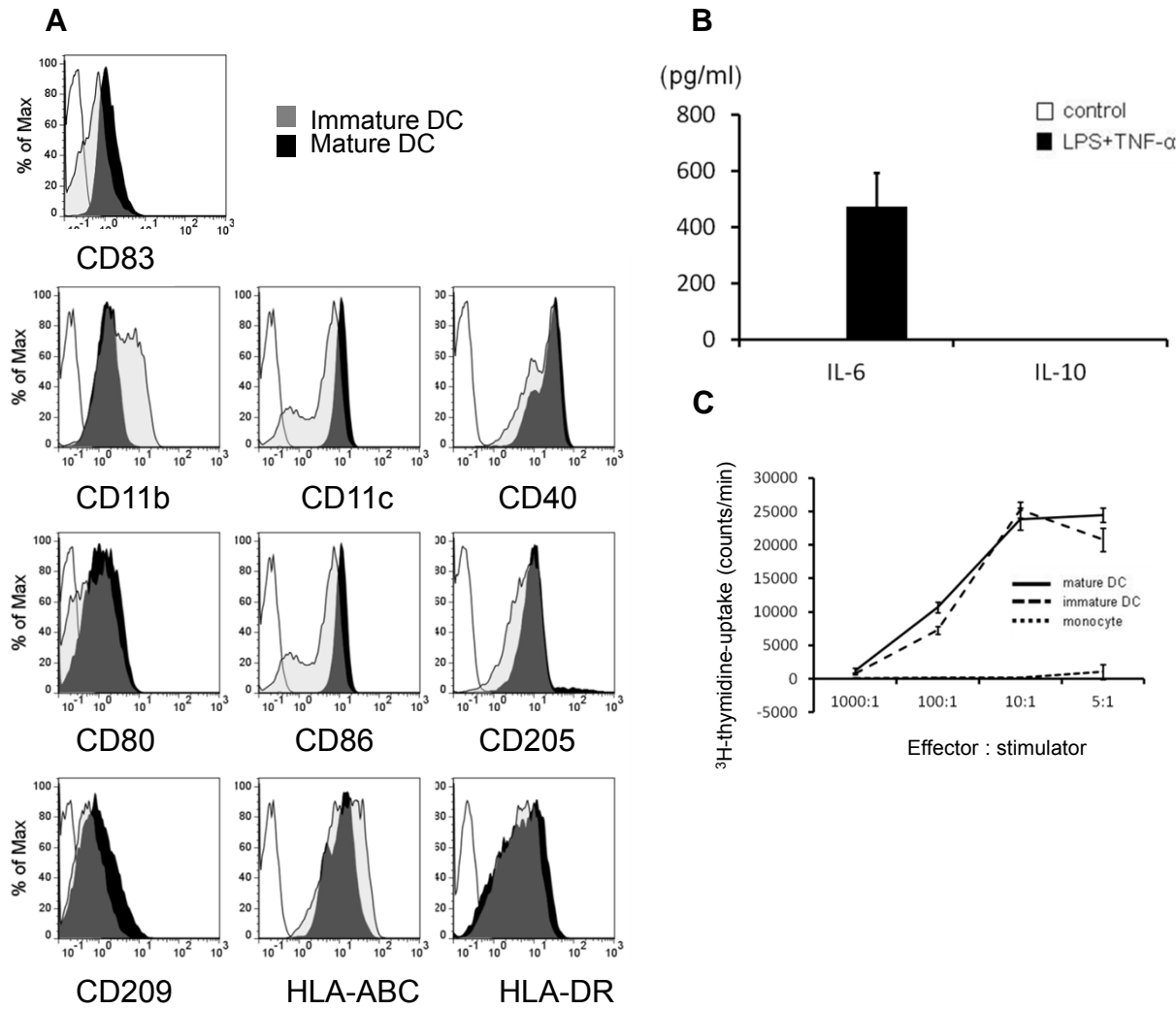

Supplement: Figure S4 — Characteristics and functional assays of dendritic cells derived from primary monocytes. (A) Flow cytometric analysis of immature/mature DCs derived from primary monocytes. (B) The levels of IL-10 and TNF-α in supernatants of culture medium with primary-DCs 24 hours after LPS stimulation. (C) The proliferation of allogeneic naïve T cells (1×10^5^ cells per well) co-cultured with 40 Gy-irradiated stimulator cells for 3 days was evaluated. The proliferation of naïve T cells in the last 16 hours was measured by 3H-thymidine uptake. (PDF) [file pone.0059243.s004.pdf]

Figure S5

A

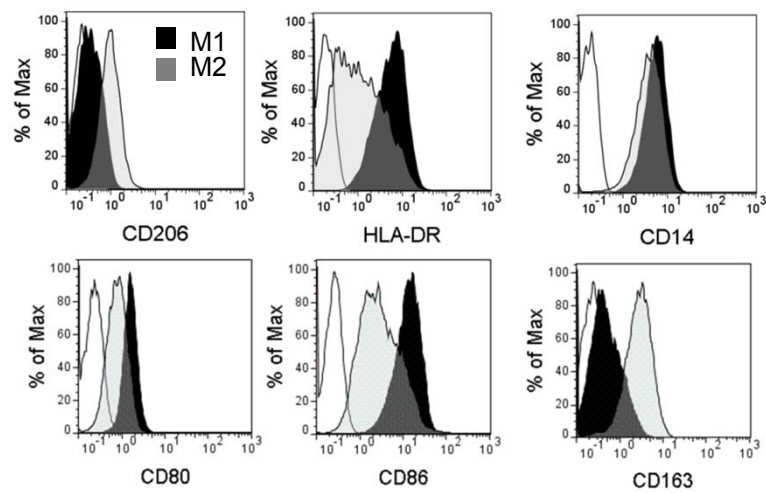

B

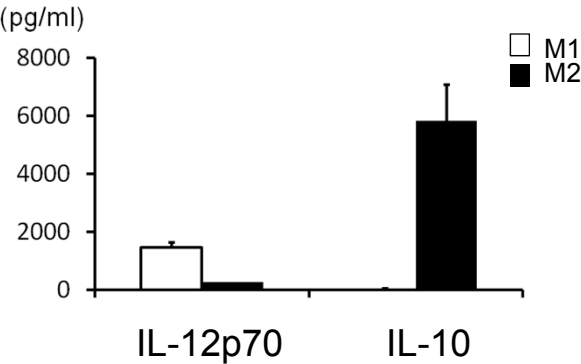

Supplement: Figure S5 — Characteristics and functional assays of M1/M2 macrophages derived from primary monocytes. (A) Flow cytometric analysis of M1/M2 macrophages derived from primary monocytes. (B) The levels of IL-12p70 and IL-10 in supernatants of culture medium with M1/M2 macrophages derived from primary monocytes 24 hours after LPS stimulation. (PDF) [file pone.0059243.s005.pdf]

### Figure S6

**A**

## Dendritic cells

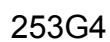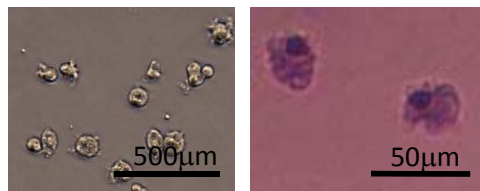

CIRA188  
Ai-W2

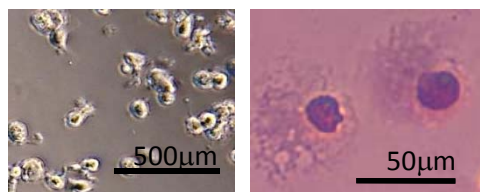

CB-A11

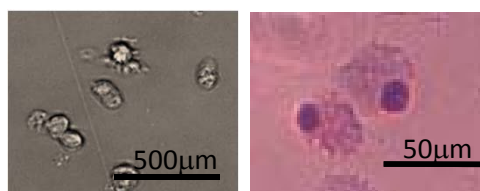

## B Macrophages

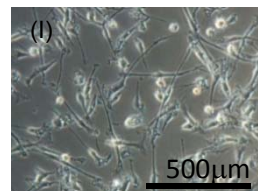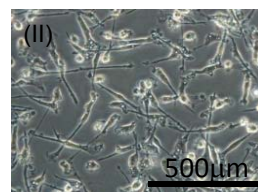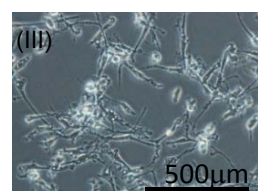

**C**

## Dendritic cells

## Macrophages

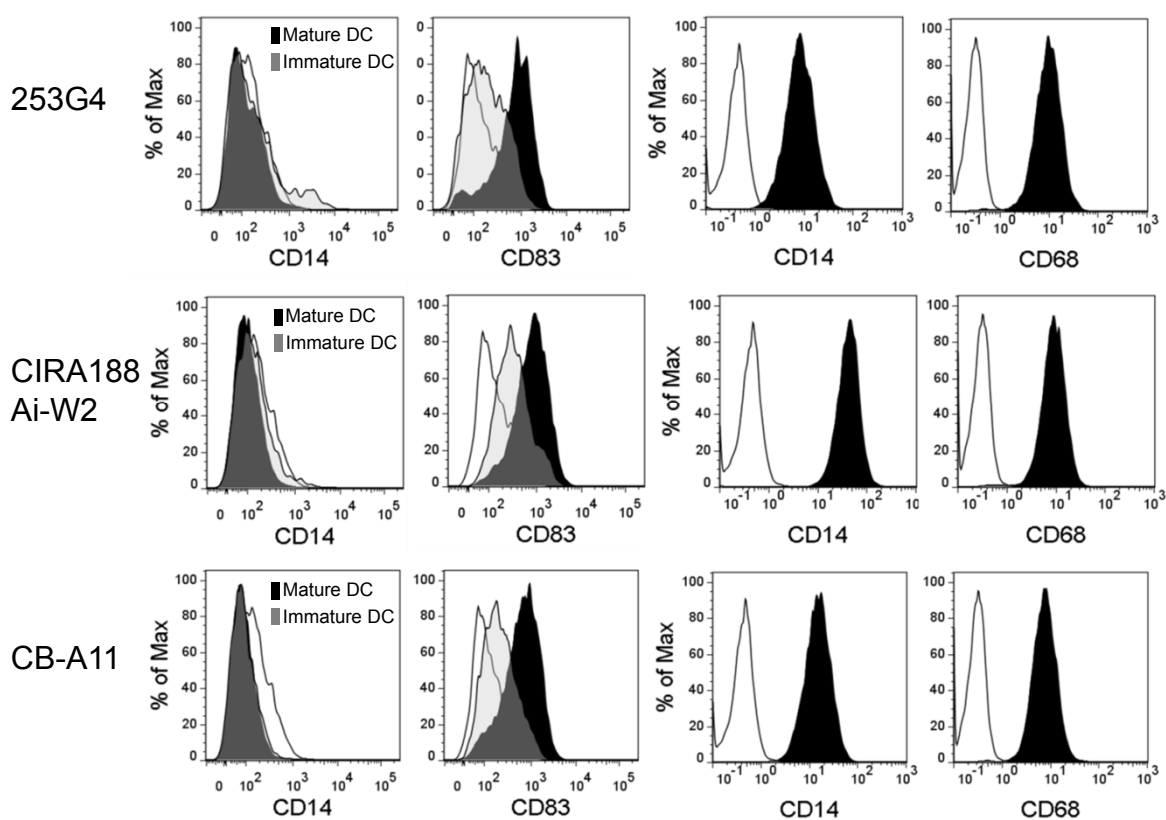

Supplement: Figure S6 — Replication assays for 3 additional pluripotent stem cell lines. (A) Phase contrast image (left) and May-Giemsa staining (right) of mature DCs derived from iPSC clones. (B) Phase contrast image of macrophages derived from iPSC clones. (C) Flow cytometric analysis of immature/mature DCs and macrophages derived from iPSC clones. (PDF) [file pone.0059243.s006.pdf]
