# Supplementary material for: Robust and Highly-Efficient Differentiation of Functional Monocytic Cells from Human Pluripotent Stem Cells under Serum- and Feeder Cell-Free Conditions
Source: PLoS One. 2013 Apr 3;8(4):e59243. doi: 10.1371/journal.pone.0059243 (PMC3616072; doi:10.1371/journal.pone.0059243)
Supplement: Table S1 — Primers for RT-PCR. (PDF) [file pone.0059243.s007.pdf]

# Table S1

| Gene | Forward | Reverse | Annealing tempature | elongation | cycle |
|---|---|---|---|---|---|
| CCL17 | ATGGCCCCACTGAAGATGCTT | TGAACACCAACGGTGGAGGT | 55 | 30 | 30 |
| CCL18 | CCCAGCTCACTCTGACCACT | CAGGCATTCAGCTTCAGGTC | 55 | 30 | 30 |
| TLR4 | GCATGGAGCTGAATTTCTAC | TTCTTTAAATGCACCTGGTT | 55 | 40 | 35 |
| PU.1 | TGGAAGGGTTTCCCCTCGTC | TGCTGTCCTTCATGTCGCCG | 62 | 30 | 30 |
| cMaf | TGCACTTCGACGACCGCTTCTC | GGTGGCTAGCTGGAATCG | 55 | 40 | 35 |
| GAPDH | ACCACAGTCCATGCCATCAC | TCCACCACCCTGTTGCTGTA | 55 | 30 | 30 |
